# Gendered pathways to youth livelihoods through sport: evidence from Fiji

**DOI:** 10.3389/fspor.2026.1855393

**Published:** 2026-07-07

**Authors:** Thomas Wanner, Katja Siefken

**Affiliations:** 1 School of Society & Culture, Adelaide University, Adelaide, SA, Australia; 2 Institute of Interdisciplinary Exercise Science and Sports Medicine (IIES), MSH Medical School Hamburg, Hamburg, Germany

**Keywords:** gender inequality, Pacific islands, socio-environmental factors, sport-for-development (SFD), sustainable livelihoods

## Abstract

**Introduction:**

Sport is frequently promoted as a mechanism for youth development and livelihood enhancement, yet empirical evidence on how sport contributes to sustainable livelihoods, particularly for young women in Pacific Island contexts, remains limited. This study examines how sport shapes livelihood opportunities for young people in Suva, Fiji, with a particular focus on gendered participation and outcomes.

**Methods:**

Drawing on the Sustainable Livelihood Framework (SLF), this qualitative case study explored how socio-environmental factors, including family dynamics, peer relations, and governance structures, influence youth engagement in sport and access to livelihood assets. Data were collected through participant observation, focus groups, and semi-structured interviews across two less-studied sports (Australian Football League and cricket), involving both active participants and those who had dropped out of sport.

**Results:**

Youth vulnerability was shaped by unemployment, fractured family relationships, and peer pressure. Sport contributed to the accumulation of human, social, and cultural capital (*vanua*), but access to these benefits was uneven. Female participants faced distinct socio-cultural and economic barriers, including domestic responsibilities, limited financial resources, and restrictive gender norms, which constrained both participation and livelihood outcomes.

**Discussion:**

The findings suggest that sport functions as a gendered livelihood pathway rather than a neutral development mechanism. Enhancing the livelihood potential of sport requires addressing the socio-environmental constraints affecting female participation through gender-sensitive sport governance, improved access to resources, and more inclusive physical education systems.

## 1 Introduction

In 2015, all member states of the United Nations agreed to Agenda 2030 and the Sustainable Development Goals (SDGs) to address global challenges like poverty, the climate crisis and social inequalities (1). The resolution highlighted the critical role of sport for achieving the SDGs, recognizing sport's contribution to sustainable development and peace, empowerment of women and young people, health, education and social inclusion. In 2018, “Sport as enabler of sustainable development” was formally enshrined in UN General Assembly Resolution A/73/L.36 which also commissioned a regular review of the progress of sport for SDGs (2). The mechanisms through which sport enables sustainable development and for whom remain underexamined, particularly in Pacific contexts where sport occupies central cultural significance (3, 4). There is also a long-running debate in the scholarship about sport-for-development whether sport essentially reproduces established social relations and inequalities or can contribute to more fundamental social change addressing social inequalities (5–7).

In this context, our research project examined the role of sport as an “enabler” for sustainable livelihoods of young people in Fiji with a specific focus on inequalities and gender. We aimed to uncover underlying barriers and facilitators that impact sports engagement among genders, thereby contributing to a more nuanced understanding of gender equity in Fijian sports. Employing the Sustainable Livelihoods Framework (SLF) as the theoretical and analytical tool, the study aimed to answer the following research questions: (1) What constitutes the vulnerability context of young sport participants? (2) How do transforming structures influence livelihood assets and vulnerability? (3) Does sport assist livelihood outcome achievement? (4) How does gender shape vulnerabilities and strategies?

Fiji was chosen as case study for examining the nexus of youth, gender, sport and livelihoods because all these factors play out in its social and cultural and development context. Fiji is an upper-middle income country which has seen progress in human development, yet there are still high levels of poverty with 29.9% of the population living below the poverty line in 2019 with high disparities between urban areas and rural areas (8). Sport, particularly rugby, plays a vital role in the social and cultural fabric of its society and as a pathway for social mobility (9).

Fiji has a high population of young people. Young people are officially defined as individuals between the ages of 15 and 35 years old, reflecting the country's specific social context. In the latest census in 2017, young people made up 33.9% of the population (10). Gender inequalities and social and economic disadvantages for young girls and women permeate society (11). Gender-based violence remains a major problem with 64% of women in Fiji having experienced violence compared to global average of 34% (8). In the 2024 Global Gender Gap Report Fiji ranks only at 128 out of 146 countries with significant gaps in economic participation, education attainment, health and political empowerment for women (12).

The paper will discuss relevant literature about sport, youth, gender and livelihoods, then outline the theoretical framework and methodology which is followed by the findings and a discussion on how sport can be harnessed as enabler for youth livelihoods and improving gender inequalities. We explore how sport may function as a livelihood pathway, and how access to and returns from sport are structured by gender and institutional context.

## Background

### Sport and (sustainable) development

Through its Sport for Development and Peace (SDP) agenda, the United Nations has long recognized the important role of sport for fostering social, cultural and economic development and promoting peace. The international development framework from 2015 to 2030, Agenda 2030, highlights the critical role of sport as an “enabler” for achieving the SDGs.

Sport-for-development (SFD) has become a vast scholarly area of research examining how sport-based initiatives contribute to social, educational and economic outcomes (6, 13, 14). While sport is often associated with benefits such as social capital, life skills and community engagement (13, 15), evidence suggests that positive outcomes are neither automatic nor universal. Coalter (5, 16) demonstrates that outcomes depend heavily on program design and contextual factors. Darnell (6) and Darnell and Hayhurst (17) employ postcolonial feminist frameworks to reveal how sport-for-development can perpetuate neo-colonial power relations, privileging Northern expertise while marginalizing Southern voices. Hartmann and Kwauk (7) critique sport-for-development's idealised belief for social transformation and argue that for transformative development the underlying power relations and assumptions about sport and development need to be interrogated.

Within the global development agenda, sport is increasingly framed as a contributor to broader socio-economic and environmental objectives. The recent focus on the role of sport for achieving the SDGs reflects the global sustainable development agenda which is inclusive of environmental, social and economic sustainability goals, and peace and international cooperation (SDG 16) (18–21).

However, much of the literature has focused on health, education and psychosocial outcomes, with comparatively limited attention to sport as a pathway to economic participation and long-term livelihood security. Empirical evidence on how engagement in sport translates into employment opportunities, income generation or career mobility remains fragmented and context specific.

### Youth, sport and SDGs

This gap is particularly evident in research on young people. Youth (aged 15–24) represent a large and growing share of the population in many low- and middle-income countries (22) yet they often face constrained access to labour markets and decision-making processes (23, 24). These patterns manifest acutely in Pacific contexts, where age-based hierarchies structure social relations, positioning youth subordinately relative to elders (25).

Sport does play a crucial role in the development of youth, serving as a powerful tool for fostering physical health, social skills, and personal growth (26). Engagement in sports activities provides young people with opportunities to develop discipline, teamwork, leadership, and resilience. Sport can serve as a pathway for social mobility, offering scholarships and career opportunities, particularly in professional sports (27). However, despite these benefits, there are stark disparities in sports participation influenced by socio-economic status and gender, necessitating reforms to ensure inclusive access (28). Understanding whether and how sport contributes to sustainable livelihood trajectories for different groups of young people therefore requires contextually grounded analysis.

The SDGs present an ambitious global agenda for social and human development with 11 of 17 goals directly mentioning youth (29). Sport's role as an “enabler” for SDGs and livelihoods of young people remains under researched and requires empirical examination grounded in local contexts.

### Sport, gender and development

Gender constitutes a critical fault line in sport-for-development. While programs increasingly target girls and women (30, 31), research reveals persistent barriers to equitable participation and benefit distribution (32). Pacific scholarship documents how rugby embodies hegemonic masculinity in Fiji, making women's sport participation transgressive (4, 33). Sport can have a positive effect for women and girls by fostering the development of a strong sense of self, social bonds, a positive body image and a sense of agency (34).

As a recent literature review shows, SFD programs targeting women and girls can help achieve SDG5 gender equality on an individual level but lack a focus on the macro-level or overall social structural level (35). However, in highly patriarchal societies like Fiji, women's and girls' participation in sport can expose them to increased risks of gender-based violence due to cultural and structural norms that resist female involvement in traditionally male-dominated spaces. Research on how SFD plays a role in improving the lives for these girls and women is still lacking, with critical researchers calling for more in-depth and larger-scale studies (34).

### Sport governance and development

Governance, the systems and processes of decision-making in organizations or societies, have become a prominent feature in development discourse because transparent, participatory and accountable forms of governance, or “good governance”, are seen as critical for achieving human development and sustainable development outcomes (36, 37). Sport governance is the theory and practice of governance applied to the sport context (38). Sport governance as a distinct field has a very short history starting this century with increasing interest in how sport organizations function and with what social, cultural and environmental impacts (39). However, analyses of sport governance in the sport-for-development field remain peripheral (40).

How sport governance operates within specific local contexts to either constrain or enhance livelihood opportunities for groups, such as young people, women, and girls, represents a critical dimension of the Sustainable Livelihood Framework's (SLF) central component “transforming structures and processes” as they shape livelihood contexts and capabilities.

## Theoretical framework: adapting the SLF for sport

**Figure 1:**
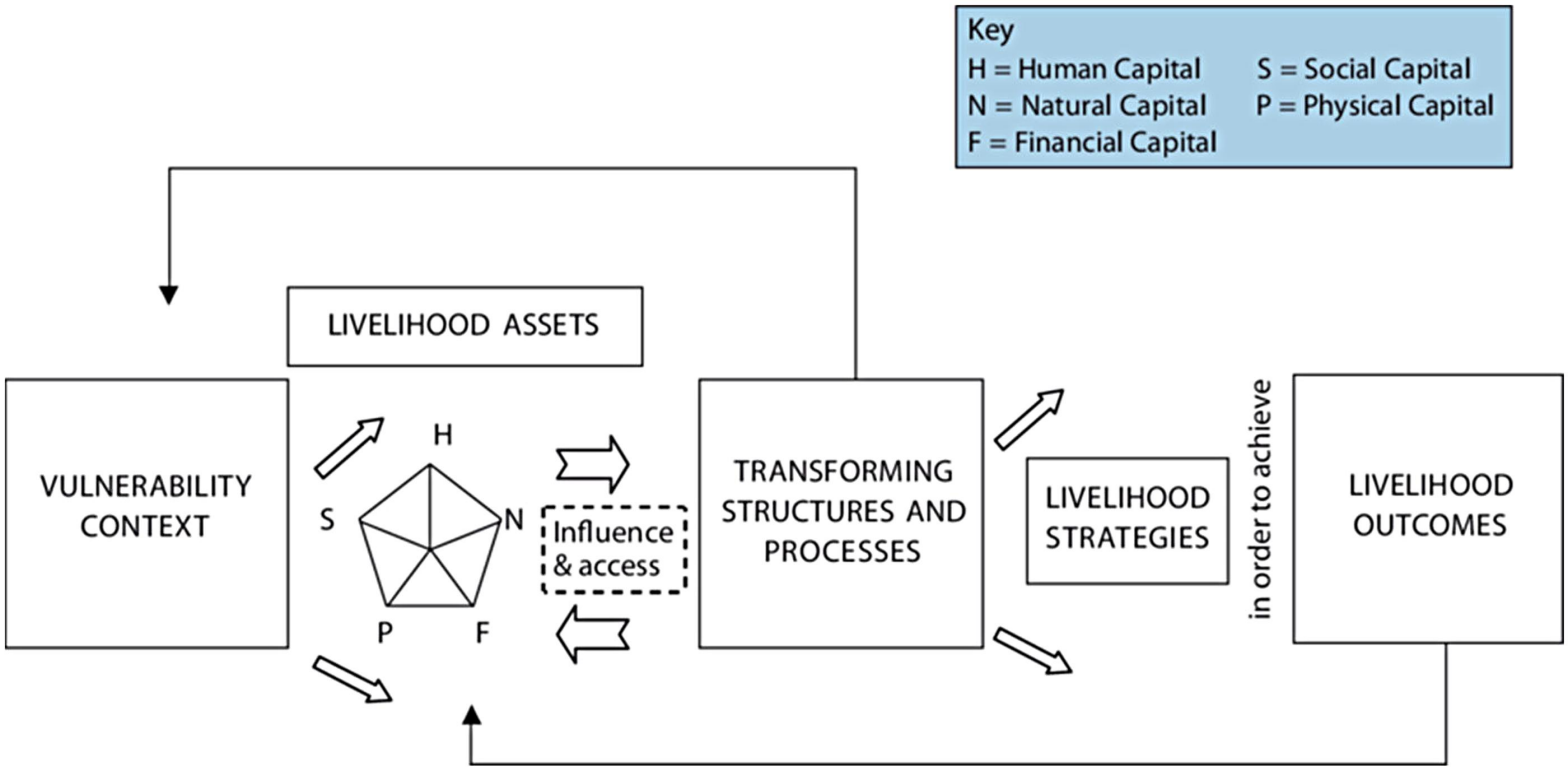
Sustainable livelihoods framework. [Source (43), n.p.].

Livelihood approaches emerged as alternatives to narrow income-focused development models, recognizing that people pursue diverse outcomes using multiple, interacting assets (41, 42). We utilized the Sustainable Livelihoods Framework (SLF), first devised by the UK Department of International Development (43), as a tool to analyze the context of poverty of communities and households by assessing their vulnerabilities, assets and capacities to achieve sustainable livelihood outcomes such as more income, food security, and better health. The focus on improvement of livelihoods of individuals, in our case young people, and of analyzing the policy and governance structures that set the conditions for improving livelihoods of young people makes the SLF a relevant theoretical and analytical framework for our research objectives (See Figure 1).

As Figure 1 shows, the SLF conceptualizes livelihoods as comprising assets of individuals or communities shaped by vulnerability contexts and mediated through transforming structures and processes which are the institutions and policies determining the assets and vulnerability context (43). Livelihood assets are the resources people have at their disposal to improve their lives. They consist of human capital (e.g., skills, knowledge, labour capacity, health), social capital (e.g., social networks, norms, relationships of trust), physical capital (e.g., transport, shelter, technology), financial capital (e.g., income, savings), and natural capital (e.g., natural resources such as land and water) (43), (p. 3).

Transforming structures and processes encompass both public and private sectors and can include policy, laws, and cultural norms (43). In the context of sport-for-development, structures may involve non-governmental organizations (NGOs), regional and national sporting federations, local sporting organizations, relevant government ministries, and family units. For this research, the role of transforming structures and processes is primarily examined through the lens of sport governance which means the mechanisms and structures of power and decision-making about sport, young people and gender in Fiji.

The SLF's non-linear conception recognizes feedback loops: assets influence access to transforming structures which shape vulnerability and enable asset accumulation (43). Power permeates these relationships with those with greater assets wielding more influence over governance structures while governance access facilitates asset accumulation, creating potential for virtuous or vicious cycles.

The SLF's analytical value lies in its dual emphasis on individual agency and structural constraints. Despite livelihoods being frequently cited in sport-for-development literature, Schulenkorf et al.'s (13) systematic review found minimal research employing livelihood frameworks. For example, Lorenzo et al. (44) explored the impact of sport and leisure on young people with a disability, finding family support to be an important “anchor” to improved livelihood outcomes. Stewart-Withers et al.'s (27) analysis of iTaukei rugby players' sport-based migration as a livelihood strategy, which revealed gender-specific patterns in how sport enables asset accumulation and mobility. Overall, however, the intersections of livelihoods and SFD continue to receive limited attention (45).

There have, of course, been critiques of the SLF that are important to acknowledge, particularly in a Pacific context. The most persistent critique is its depoliticisation of poverty and development: by framing structural inequalities as manageable “structures and processes”, the framework tends to obscure how power relations, class dynamics, and political economy actively reproduce disadvantage (46, 47). Related to this, the SLF has been criticised for what Bebbington (48) influentially termed “asset fetishism” which is a tendency to reduce livelihoods to capital accumulation while overlooking meaning, dignity, and social relations. This limitation is particularly consequential for sport-for-development research, where symbolic, cultural, and relational dimensions of participation matter as much as material outcomes. The SLF has also been critiqued for its gender-neutrality. Despite its utility as an analytical tool, it was not designed with a feminist or intersectional lens and tends to treat the household as a unified unit of analysis, thereby masking intra-household inequalities, including gendered control over assets, decision-making, and the distribution of benefits from participation (49, 50). While the SLF provides the primary framework for our examination of vulnerabilities, assets, and livelihood outcomes, we also draw on insights from feminist sport scholarship e.g., (51, 52) to better understand how gendered power relations shape access to resources, opportunities, and livelihood pathways. We also highlight that gender in itself is a major dimension shaping the vulnerabilities and livelihoods context of young people in Fiji.

We modified the standard five capitals by excluding “natural capital” (environmental resources like land because young people do not generally have this as an “asset” at their disposal) and replaced it with cultural capital or *vanua*, reflecting its documented importance in Fijian contexts (53, 54). *Vanua* translates literally as “land” but encompasses interconnected concepts of family, tradition, community, and pride, organizing Fijian social hierarchies based on age, gender, and rank (55, 56). Sport participation, particularly representing Fiji internationally, generates *vanua*, making this cultural capital form central to understanding sport as a livelihood strategy.

## Methods

### Research design and setting

This study employed a qualitative case study to explore the relationship between youth sport participation and livelihood opportunities in Suva, Fiji. Data were collected through participant observation, focus groups, and semi-structured interviews. This allowed an in-depth examination of young people's lived experiences within their socio-cultural context. The study followed the Consolidated Criteria for Reporting Qualitative Research (COREQ) guidelines to enhance transparency and rigor (57). Ethical approval was obtained from the University of Adelaide Human Research Ethics Committee (H-2019-016).

### Positionality

As researchers, we occupy particular social and institutional positions that shape both the questions we ask and the knowledge we produce. We are Western-educated academics, one male and one female, conducting research with young Fijians on livelihoods, opportunity, and the role of sport in their lives. Our positionality carries important epistemological and ethical implications. Our social, institutional, and geographic location places us within a long tradition of Northern researchers studying communities in the Global South, a tradition deeply entangled with colonial knowledge production and the power asymmetries it continues to reproduce (58, 59). We therefore sought to remain reflexive throughout the research process regarding how our assumptions, relationships with participants, and interpretive choices may have shaped the research.

Our gender difference as a research team offered some methodological advantages particularly in creating space for young women and young men to speak more freely in certain settings but gender alone does not resolve the more fundamental asymmetries of race, class, and institutional power that positioned us as outsiders in the communities we worked with. We were conscious throughout of the risk of reproducing what Spivak (60) identified as the problem of speaking *for* rather than *with* marginalised communities, and we sought to mitigate this through prolonged engagement, member checking, and ongoing dialogue with local partners and community gatekeepers. Nonetheless, we acknowledge that our interpretations remain partial and situated, and that Indigenous Fijian perspectives including those grounded in *vanua* and relational ways of knowing exceed what our analytical frameworks, however critically adapted, are fully able to capture (54).

### Participants and recruitment

*Participants* were recruited using purposive sampling to capture diverse perspective across sport participation, gender, and socio-economic backgrounds. The sample included young athletes (aged 15–30 yrs.), individuals who had dropped out of sport as well as key stakeholders such as coaches and sport administrators. The sample comprised 45 participants, with approximately equal representation of males and females. The study focused on Australian Football League (AFL) and cricket because these sports remain comparatively under-researched within the Fijian context, where research has predominantly focused on rugby. Both sports have established grassroots development programmes and have actively sought to increase female participation, making them particularly relevant for examining gendered livelihood pathways. The intention was not to provide a representative assessment of all sports in Fiji but rather to generate in-depth insights from two contrasting sporting contexts that have received limited scholarly attention.

## Data collection

Participant observation was conducted at sporting events and training sessions to capture everyday practices, interactions, and contextual dynamics. Detailed field notes were recorded throughout.

Three focus groups (4–6 participants each) were conducted: one with female participants and drop-outs, one with male participants and drop-outs, and one with sport administrators. Discussions were facilitated using a Talanoa approach, consistent with iTaukei cultural practices that emphasise open, relational dialogue (61, 62).

In addition, 38 semi-structured interviews were conducted with athletes, drop-outs, coaches, and administrators. Interview questions were guided by the Sustainable Livelihood Framework and explored vulnerability contexts, livelihood assets, gendered experiences, and perceived outcomes of sport participation. Female participants were interviewed by local female research assistants to enhance comfort and openness. Table 1 presents examples of interview topics rather than the complete interview guide. Interviews were conducted in a semi-structured format, with follow-up questions used to encourage participants to elaborate on their experiences and perspectives.

**Table 1:** Overview of interview questions.

Sustainable Livelihood Framework	Sample Questions
Livelihood Context	What are the major challenges young people face in Fiji?
Livelihood Assets	
Human capital	Why do/did you participate in sport?
	Why did you stop playing sport?
Social capital	In what ways, if any, do teammates support you outside of sport?
	How, if at all, has participation in sport influenced your access to employment opportunities or professional networks?
Cultural capital	What are the cultural factors that motivate people to participate in sport?
	What role does sport play in creating pride or recognition within your family, community, or village?
Financial capital	How has money effected your participation in sport?
Physical capital	How would you describe the facilities and resources available to your team?
Livelihood Strategies	What would you like to do in the future?
	How has sport assisted in achieving your plans?
Transforming Structures and Processes	How does your organization contribute to the success of grassroots sport?
Values	What values does sport instill?
	What do young people want to get out of sport?
Inclusiveness and Gender	How do you ensure inclusiveness in sport regardless of playing ability at grassroots sports clubs?
	What role does your organization play in promoting gender equality in sport?
	What are the major challenges for girls and women/boys and men when playing sport in Fiji?
Livelihood Outcomes	How did/does sport contribute to your life?
	In what ways has sport influenced your future plans and opportunities?

All participants provided informed consent, with parental consent obtained for minors. Participants were anonymized using identification codes. Documentary analysis of policy documents related to sport, education, youth and gender in Fiji complemented primary data and informed the analysis of governance structures. The documentary review included national policy documents relating to physical education and school sport, and national gender policies. The documents were reviewed to identify policy priorities, governance arrangements, and stated commitments relating to youth participation, gender equality, and sport development. Rather than serving as a primary source of empirical data, the documents were used to contextualise participant perspectives and inform the analysis of transforming structures and processes within the Sustainable Livelihood Framework.

In all instances, female sport participants and drop-outs of sport were interviewed by local female research assistants to enhance participant comfort and encourage open dialogue. Each participant was assigned a unique identification number, and the data presented in this study is attributed to this number, along with two additional characteristics: gender and the type of sport in which they participated.

## Data analysis

All data were transcribed verbatim and analyzed using reflexive thematic analysis following Braun and Clarke (63, 64). Analysis involved iterative coding, theme development, and refinement across the dataset. NVivo software supported data organization and coding. Particular attention was given to identifying patterns related to gendered participation, access to resources, and the role of sport in shaping livelihood opportunities.

## Findings

### Vulnerability context: challenges facing young Fijian sport participants

#### Unemployment and economic precarity

Youth unemployment emerged as the most frequently cited vulnerability, reflecting national patterns where 18.5% of 15–24 year-olds are unemployed (65). Participants described intense pressure to secure employment and contribute financially to extended families, pressure heightened by traditional expectations that young men particularly should provide for relatives. An AFL administrator articulated: “These kids, they're under so much pressure from their families. If you're not working, you're seen as a failure”.

Unemployment intersected with educational challenges. Many iTaukei participants attended school primarily to maintain sport participation, viewing education instrumentally rather than intrinsically valuable. When asked about future plans, a male cricket player responded: “I just want to get a contract [in professional sport]. School is just something I do until then”.

Economic vulnerability particularly affected those in informal settlements. Approximately 60% of participants resided in squatter settlements surrounding Suva, where families had migrated from rural areas seeking educational and employment opportunities. Settlement life brought proximity to services but also precarity such as insecure land tenure, limited infrastructure, and concentrated poverty.

#### Fractured family relationships

Family relationships constituted the second major vulnerability dimension, characterized by separation/divorce, domestic violence, and tensions around traditional authority structures. A male AFL player who also worked as a development officer explained: “I was brought up from a broken family. And then I went through a lot of shit. So every time I come to train … it takes my mind off everything”.

The *Vaka i Taukei* (Fijian way of life) structures family relationships around age and gender hierarchies wherein youth occupy subordinate positions, expected to demonstrate obedience to elders, with binary labeling whereby youth are categorized as either compliant Christians or deviant “heathens” (*tamata butobuto*) (25, 56).

For young men, family pressure centered on sport achievement. In families where relatives had represented Fiji in rugby or other sports, expectations that current generation youth would replicate these achievements created immense stress. Young women faced different but equally constraining family pressures. Participants described being expected to perform domestic labour (cooking, cleaning, childcare) before and after school/work, limiting their time for sport. A female AFL administrator explained: “With women, one of the challenges, you have to clean the house and if that is done you can come … you have to prepare breakfast, clean the dishes … That's why girls it can be difficult to come to training”. Some female participants hid their sport participation from families, aware that contact sports like rugby and AFL were considered unfeminine and inappropriate.

#### Peer pressure and housing

Peer pressure constituted the third vulnerability dimension, manifesting distinctly for males vs. females and varying by residence. For young men in settlements, peer pressure typically involved older dropout youth encouraging substance use (marijuana, alcohol, glue sniffing) and petty crime. For females, peer pressure operated differently, often discouraging sport participation rather than encouraging deviant behavior. Young women described ridicule from male peers about their bodies, athletic ability, and sexuality, with female rugby players particularly facing assumptions about being lesbian. A male cricket administrator reflected: “When I walked in they were the joke of the cricket fraternity, especially amongst the men, the men were making fun saying ‘how is your sumo team’”. Such ridicule, whether direct or overheard, deterred many females from sport.

## Livelihood assets: how sport enables asset accumulation

### Human capital: skills, knowledge, and health

Sport participation facilitated human capital development through multiple pathways. Most directly, participants acquired sport-specific skills and knowledge transferable to coaching, refereeing, and sport administration roles. Several participants had progressed from players to sport development officers, earning modest incomes teaching sport in schools, one of few formal employment pathways available to youth without tertiary education.

Beyond sport-specific skills, participants described “life skills” learned through sport. A female AFL player explained: “Sport teaches you discipline, time management, respect for others”. Coaches and administrators explicitly used sport as a vehicle for conveying broader lessons about teamwork, leadership, and perseverance.

However, human capital development faced constraints. Physical education in schools emphasized competitive sport over foundational movement skills or health education, meaning many participants could play specific sports but lacked broader physical literacy or health knowledge.

## Social capital: networks, relationships, and trust

Social capital accumulation emerged as sport's most prominent benefit. Participants consistently emphasized friendships, networks, and sense of belonging generated through sport. A male cricket player stated simply: “The best thing about cricket is the friends I've made”. These relationships provided emotional support, information about opportunities, and practical assistance navigating challenges.

Sport-generated social capital had bonding and bridging dimensions (66). Bonding capital strengthened within-group ties like teammates developing deep trust and solidarity through shared experiences. A female AFL player described how teammates had become “like sisters”, providing support during family difficulties. Bridging capital connected participants across social divides with settlement residents playing alongside middle-class youth, iTaukei and Indo-Fijians cooperating toward shared goals.

Network benefits extended beyond sport. Sport contacts helped participants access employment information, educational opportunities, and civic engagement. Administrators served as mentors, writing recommendation letters and making introductions. Several participants secured employment through administrator networks, illustrating how sport-generated social capital converted to economic opportunities.

However, social capital accumulation was gendered and conditional. Males more readily converted sport social capital into tangible opportunities, particularly employment. Females built networks but struggled translating these into career pathways, reflecting broader gender barriers in Fijian society.

## Physical capital: equipment, facilities, and infrastructure

Sport participation required physical capital in form of equipment, facilities, and transportation creating access barriers for economically vulnerable youth. While national federations subsidized equipment for development programs, participants often lacked personal equipment for practice. Facility access posed greater challenges, with most fields privately owned by schools or controlled by government agencies charging rental fees.

Transportation constituted a critical physical capital need, particularly for participants from outlying settlements. Training and matches occurred across Suva requiring bus trips costing several dollars each way which is a substantial amount for unemployed youth. Female participants particularly cited transportation as a participation barrier, as safety concerns about traveling alone after dark limited their mobility.

## Financial capital: income, savings, and assets

Financial capital remained the scarcest asset for most participants. While sport participation sometimes generated income through development officer wages, referee fees, or prize money, these remained modest and irregular. A male AFL development officer earned approximately $100 USD weekly conducting school clinics, supplementing family income but insufficient for independence.

More significantly, sport opened pathways to financial capital through indirect mechanisms. For males, representing Fiji in national teams created eligibility for police or army employment. Several AFL players described explicitly pursuing sport excellence to access these employment pathways. One stated: “If I can make the Fiji team, I can get into the army. That's my plan”.

However, these pathways were profoundly gendered. While elite male athletes could leverage sport achievements for military/police employment, no equivalent pathway existed for females. Female athletes of similar caliber worked as development officers earning half what their male counterparts earned in uniformed services.

## Cultural capital and *vanua*: pride, prestige, and belonging

Cultural capital accumulation, particularly *vanua*, emerged as a central livelihood outcome. *Vanua* encompasses interconnected concepts of family, land, tradition, community, and pride, organizing Fijian social hierarchies (55, 56). Representing one's village, province, or nation in sport generated *vanua*, elevating participants' social standing.

This was evident in how administrators introduced participants: “This is [Name], he played for Fiji in the International Cup”. Such introductions immediately established credibility and respect. Social media posts about sport achievements garnered extensive community engagement, with relatives sharing and celebrating. A male AFL player from an informal settlement explained: “Where I come from, people know me because of football. It makes my family proud”.

*Vanua* accumulation motivated international travel aspirations beyond financial considerations. Participants desperately wanted to represent Fiji abroad, viewing this as the ultimate honor. A female cricket player stated: “My dream is to play for Fiji in the World Cup. To make my community proud”. This cultural capital dimension helps explain sport's appeal despite modest financial returns, *vanua* provided social value that economic capital couldn't purchase.

However, *vanua* accumulation was fragile and gendered. It required sustained achievement; failure to maintain sport excellence led to *vanua* erosion. Female athletes accumulated less *vanua* than male counterparts for equivalent achievements, reflecting gendered prestige valuations in Fijian society. One female administrator noted: “When the men's team wins, the whole country celebrates. When we win, nobody notices”.

## Transforming structures and processes: governance and constraints

### National sport federations: resources and limitations

National Sport Federations (NSFs), AFL Fiji and Fiji Cricket, constituted primary transforming structures, organizing competitions, delivering development programs, and managing resources. Both federations operated as non-profit entities with volunteer boards, small paid staff, and heavy dependence on international funding.

Administrators described constant resource constraints, forcing difficult prioritization between grassroots participation programs and elite athlete development. A cricket administrator explained: “We want to support everyone, but we have to focus resources where we can qualify for international tournaments. That brings in more funding”. This logic created a pyramid structure prioritizing elite performance, with fewer resources allocated to mass participation despite rhetoric about “sport for all”.

Governance structures excluded youth voices despite young people comprising the majority of participants. Boards consisted of older, established community members like former players, business people, government officials. No NSF had youth representatives despite proposals to create youth advisory bodies.

Both NSFs demonstrated capacity constraints in areas like strategic planning, monitoring and evaluation, and stakeholder coordination. Gender representation in NSF governance varied. Cricket had more female administrators than AFL, including senior positions, though men still dominated executive roles.

### Government: ministries, policies, and priorities

Government engagement with sport occurred through multiple agencies with overlapping mandates. The Ministry of Youth and Sport developed policies and allocated funding; the Fiji National Sport Commission (FNSC) distributed grants and coordinated international sport-for-development programs; the Fiji Sports Council (FSC) managed facilities.

Sport policy emphasized elite athlete development and international competitiveness rather than mass participation or livelihood development. The National Sport Policy articulated objectives around international sporting success, positioning sport as a vehicle for national pride. References to sport and development focused on infrastructure (facilities, coaching) rather than holistic development.

Funding allocations reflected elite priorities. NSFs received grants based partially on international competitive success, creating incentives for elite focus. Rugby received by far the largest allocations, reflecting its cultural significance and international success.

Policy implementation gaps between stated commitments and practice were evident. The National Sport Policy emphasized gender equality and youth development but these remained rhetorical without concrete targets, budgets, or accountability mechanisms. No systematic monitoring occurred regarding youth sport participation rates, dropout patterns, or barriers faced by marginalized groups.

### Schools: physical education and competitive sport

Schools constituted critical structures for youth sport participation. The dominant PE model prioritized sport-specific skills and competitive performance over foundational movement literacy or health education. NSF development officers regularly conducted school clinics teaching cricket or AFL skills, then organizing competitions to identify talent for representative pathways.

This approach aligned with the pyramid model of sport development, positioning schools as talent identification venues rather than contexts for mass physical literacy development (67). While it served elite athlete pathways, it failed many students. Those deemed “not talented” often withdrew from PE, denied opportunities to develop movement competence and health knowledge valuable regardless of athletic talent.

Competitive sport emphasis also reinforced gender inequalities. Schools invested resources in boys' sports, particularly rugby, while providing minimal support for girls' sports. Some schools explicitly prohibited girls from playing contact sports like rugby or AFL despite national curriculum policies promoting gender equity.

### International sport organizations: resources and power asymmetries

International sport organizations profoundly shaped Fijian sport through funding, governance structures, and competitive pathways. AFL Australia and the International Cricket Council (ICC) wielded enormous influence through their control of resources and rule-making authority. Both organizations operated development programs in Fiji providing equipment, training, and competition opportunities. However, these relationships exhibited neo-colonial patterns wherein Northern organizations maintained power while ostensibly supporting Southern development (6).

Cricket governance exhibited neo-colonial patterns. The ICC includes Fiji as an Associate Member, nominally providing governance voice. However, the ICC's structure prioritizes Full Members, the twelve nations controlling decision-making and receiving most of the revenue from broadcast rights, despite Associate Members comprising over 90 nations. A cricket administrator explained: “We're members but we don't have a real say. The big countries decide everything”.

Funding models perpetuated dependency. Both sports received development grants from international bodies but these came with conditions requiring alignment with Northern priorities. AFL funding emphasized growing Australian football's international footprint, positioning Fiji as a market for Australian sport rather than a partner in mutual development.

### Livelihood outcomes: what youth want from sport

Analysis revealed three primary livelihood outcomes motivating sport participation: improved income, spaces for youth, and cultural capital/*vanua* accumulation.

### Improved income: professional contracts and employment

Income generation motivated most male participants and many females, though gendered pathways differed markedly. Males consistently articulated aspirations for professional contracts: “Everyone's dream is just to secure a contract” which represent sport as a pathway out of poverty.

However, professional contracts remained statistically improbable. While Fiji produces professional rugby players regularly, few AFL or cricket players secure overseas professional opportunities. For the hundreds participating in grassroots programs, professional sport represented an unlikely outcome. Administrators described this as a challenge: “They all want to be professionals, but we can only send one or two. Most will never make it”.

Despite low probabilities, professional sport aspirations shaped participants' education and employment decisions. Males often delayed or declined educational opportunities, remaining in school solely to play sport, gambling on sport success rather than developing alternative skills.

For males, sport achievements could generate indirect income through security service employment even without professional contracts. Representing Fiji made individuals eligible for police or army recruitment, providing formal employment with modest but stable salaries, benefits, and prestige. Multiple players explicitly articulated this pathway: “If I can make the Fiji team, I can get into the army. That's my plan”.

Females faced different income dynamics. While some female athletes aspired to professional contracts, most recognized these were even less available for women given limited professional women's leagues globally. Instead, females often pursued sport for development officer positions, coaching roles, or educational scholarships abroad which are less lucrative but more realistic than professional playing contracts.

The gendered income disparity through sport reflected broader labor market inequality. Males could leverage sport achievements for military employment; females couldn't despite equivalent athletic ability. Males earned higher development officer salaries than females in similar roles. These sport-based inequalities mirrored and reinforced economic marginalization, making sport a less effective livelihood strategy for females.

### Spaces for youth: freedom, travel, and social connection

Youth desire for “spaces”, the physical and social contexts for youth-controlled activities, emerged as a powerful livelihood outcome. Fijian age hierarchies position youth subordinately, requiring deference to elders and limiting autonomous decision-making (25). Sport provided rare contexts where youth could socialize peer-to-peer, make decisions, and construct identities less constrained by adult supervision.

Training sessions revealed youth laughing, joking, and interacting with freedom rarely observed in other settings. They played music (Caribbean reggae popular among youth but not adults), wore casual clothes, and spoke informally, all contrasting with formal behavior required in family or church contexts. Sport created legitimate youth spaces, culturally acceptable because sport itself was valued by adults.

Travel particularly represented space creation. International tournaments enabled youth to visit Australia, New Zealand, or other Pacific nations, experiencing environments beyond Fijian adult supervision. Participants described these trips enthusiastically. Even domestic travel generated valued experiences. Participants from settlements or rural areas eagerly anticipated trips to Suva or visits to resort areas for tournaments. These trips exposed them to higher socioeconomic environments and experiences otherwise inaccessible.

Socially, sport created alternative peer networks outside family-defined relationships. Friendships formed through sport teams provided emotional support, information exchange, and social belonging particularly valuable for youth facing family difficulties. A male player explained: “When things are bad at home, I know I can come to training and my teammates are there”.

However, these spaces had limits. Youth still faced adult authority through coaches and administrators. Sport rules and discipline reflected adult priorities, sometimes constraining youth expression. Female participants particularly described how sport spaces remained masculinized through the domination of male administrators, male players' priorities, and assumptions about appropriate feminine behavior, all limiting their freedom within sport contexts.

### Cultural capital/*vanua*: pride, recognition, and belonging

Cultural capital accumulation, specifically *vanua*, motivated sport participation as significantly as economic considerations. Participants consistently articulated desires to “represent Fiji”, “make their community proud”, and “bring honor to their village”. These aspirations reflected *vanua* as a livelihood outcome: social prestige, community recognition, and elevated status within traditional hierarchies.

This manifested through community celebration of sport achievements. When participants represented Fiji internationally, their villages organized church services giving thanks, families shared news on social media, and community members discussed achievements with pride. One administrator described: “When [Name] made the Fiji team, his whole village celebrated. They had a feast, they talked about it for months”. For participants from non-chiefly backgrounds, sport success provided rare opportunities to accumulate prestige otherwise denied by birth status.

Sport-generated *vanua* sometimes converted to tangible benefits. Participants described receiving community support in form of financial contributions for equipment, food provided by relatives because of sport achievements. This support reflected *vanua* accumulation translating into reciprocal obligations, with communities investing in athletes bringing collective honor.

National team selection represented the pinnacle of *vanua* accumulation. Participants revered “Fiji reps” those who had represented the nation treating them as quasi-celebrities. At tournaments, national team players were introduced with their “rep” status prominently noted.

The cultural capital dimension helps explain sport's appeal despite modest financial returns. When asked why they pursued sport despite uncertain economic prospects, participants frequently referenced family and community pride over money. A female player stated: “Even if I never get a contract, I want to represent Fiji. That's what matters to my family”.

However, *vanua* accumulation was precarious. It required sustained performance. Former players who failed to secure professional contracts sometimes became objects of ridicule rather than respect, demonstrating cultural capital's fragility (33). Gender inequality manifested here too, with female athletes accumulating less *vanua* than males for equivalent achievements.

## Discussion

### Sport and sustainable livelihoods

Our findings demonstrate that sport functions as a livelihood enabler in Fiji generating assets, reducing vulnerabilities, and facilitating valued outcomes but its effectiveness depends heavily on gender, structural factors, and governance quality. This aligns with Coalter's (5) insistence that sport's developmental impacts are contingent rather than inherent, and with Levermore's (68) observation that the relationship between sport and development is far more complex than policy rhetoric suggests.

Sport most clearly enables livelihoods through social capital accumulation. Consistent with existing research (69, 70), participants develop networks, relationships, and trust through sport participation. This resonates with Putnam's (66) theorisation of social capital as comprising both bonding ties (within-group solidarity) and bridging ties (cross-group connections), and with Woolcock's (71) emphasis on the importance of linking social capital that connects communities to institutional resources. However, social capital remained concentrated within sport communities, rarely bridging to employment or educational sectors. As Nicholson and Hoye (72) caution, the relationship between sport participation and social capital formation is not automatic but depends on the quality of social interactions and the institutional environment in which they occur. Without intentional linkages to broader opportunity structures, sport-generated social capital has limited convertibility to other livelihood benefits.

The human capital dimension reveals contradictions. While sport develops physical skills and “life lessons”, it often displaces formal education, particularly for iTaukei males viewing sport as their primary livelihood pathway. As Haudenhuyse et al. (73) argue, the human capital benefits attributed to sport participation are often assumed rather than empirically demonstrated, particularly for marginalized youth. Sport administrators' and families' tacit support for this prioritization, valuing sport over education for talented youth, demonstrates how sport can undermine rather than enable sustainable livelihoods by narrowing young people's options. This echoes Giulianotti's (74) warning that uncritical promotion of sport risks reproducing the very inequalities development interventions seek to address.

Financial capital benefits remain modest for most participants. While elite athletes can leverage sport for professional contracts or security service employment, these pathways remain inaccessible to the vast majority. The professional contract dream functions somewhat like a lottery with the few winners as visible exemplars inspiring continued participation, while the many losers remain invisible (75). These dynamics raise ethical questions about sport-for-development organizations promoting sport as an economic pathway when most participants will not achieve meaningful financial returns. As Connell (9) demonstrates in the Pacific context, the global sports labour market is deeply stratified, and the migration aspirations it generates among young Pacific Islanders can produce precarity rather than prosperity for the majority who do not secure contracts.

Cultural capital/*vanua* accumulation represents sport's most distinctive livelihood contribution in the Fijian context. Our findings extend Stewart-Withers et al.'s (27) documentation of *vanua*'s importance among professional rugby players, demonstrating its salience even for grassroots participants. This finding is significant because it suggests that Bourdieu's (76) conceptualization of cultural capital, developed in Western European contexts, requires adaptation to account for Indigenous value systems that organise social life differently (54). Vanua provides culturally specific value that economic frameworks cannot capture, suggesting livelihood approaches must attend carefully to local value systems, as Scoones (95) and De Haan and Zoomers (77) have argued for livelihood studies more broadly. However, *vanua*'s precarity, requiring sustained performance and bringing obligations alongside benefits, complicates easy narratives about sport generating sustainable livelihoods.

### Gender: sport's reproduction of inequality

Gender inequality emerges as the most troubling finding, with sport simultaneously providing opportunities for female empowerment while reinforcing patriarchal structures. Female participants demonstrated equal commitment, ability, and aspiration compared to males, yet faced systematically worse outcomes due to structural barriers both within sport and in broader society. This dual dynamic confirms Hayhurst's (51) argument that sport-for-development programs can both empower and constrain women, depending on how they navigate existing gender regimes.

Feminist sport scholars have argued that sport is not a neutral social institution but a site where gendered power relations are reproduced, negotiated, and occasionally challenged (51, 52, 78). From this perspective, inequalities in sport participation and outcomes are shaped not only by individual choices but also by social norms, institutional arrangements, and unequal access to resources. Our findings support this interpretation. Female participants faced barriers extending beyond the sporting arena, including domestic responsibilities, financial constraints, gendered expectations, and limited access to leadership and employment opportunities. At the same time, sport provided a space through which some women developed confidence, social networks, and forms of social and cultural capital that challenged traditional expectations. Consistent with feminist sport-for-development research, these findings suggest that sport can simultaneously reproduce and contest gender inequalities depending on the broader social and institutional context in which participation occurs.

The employment pathway differential exemplifies this inequality. Males representing Fiji accessed security service employment; females didn't despite equivalent athletic achievement. This gendered opportunity structure reflects Fiji's broader labour market segregation (79). Sport participation itself doesn't challenge these patterns but accommodates and potentially legitimizes them by positioning sport success as valuable for males through employability while offering females only cultural recognition. As Messner (78) has argued, the institutional structures of sport remain deeply gendered, serving as sites where hegemonic masculinity is produced and reproduced.

Sport governance patterns reproduce gender inequality through male-dominated boards, masculinized organizational cultures, and resource allocation favouring men's sports. The absence of systematic gender disaggregated monitoring meant female-specific challenges (domestic labour expectations, safety concerns, sexualization) remained invisible to male administrators. This mirrors findings by Adriaanse & Schofield (80) on the persistent underrepresentation of women in sport governance globally, and by Saliya (81) who characterized women in sport leadership in Fiji as “pink unicorns”.

Prize money and salary disparities within sport demonstrate how sport-for-development can actively perpetuate rather than address gender inequality. Organizations positioning themselves as promoting development through sport simultaneously maintain grossly unequal compensation, sending clear messages about female athletes' relative value. This represents a form of 'sportswashing' using symbolic inclusion to deflect attention from social problems and inequalities (82).

Sport-for-development must move beyond increasing female participation to examining and addressing how sport governance, resource distribution, media representation, and cultural messaging either challenge or reinforce gender hierarchies (35). This requires confronting uncomfortable realities about sport's role in constructing masculinity (59, 83) and how sport organizations benefit from existing gendered structures. As Forde and Frisby (52) contend, genuine gender transformation in SFD demands attention to the structural and institutional dimensions of inequality, not merely individual-level participation outcomes.

## Physical education reform: from sport as competition to health promoter

Current physical education approaches priorities competitive performance over foundational development, constraining sport's developmental potential. The pyramid model identifying talent early for elite pathways (67) serves national sport federations' interests in international competitive success but fails most students. As Kirk (84) argues, the dominant model of physical education as multi-sport performance has remained remarkably resistant to change despite decades of critique and evidence of its exclusionary effects.

Our findings suggest reforming PE to emphasize foundational movement literacy and health education rather than sport-specific competitive skills. This would involve primary education focusing on fundamental movement patterns applicable across activities; secondary education introducing sport-specific skills through modified games maximizing participation; and using sport as a pedagogical tool for health education addressing non-communicable diseases, sexual and reproductive health, and substance use prevention. Such an approach aligns with Whitehead's (85) concept of physical literacy, which centres lifelong engagement in physical activity rather than competitive performance.

Such reform faces institutional resistance. National sport federations benefit from current systems providing early talent identification, though this narrows participation. Schools lack trained PE teachers and view competitive sport as motivating student engagement. Overcoming these barriers requires coordinated action across education ministries, sport federations, and health agencies with such coordination currently absent as documented in intersectoral health promotion literature (86).

The cultural concept of *vanua* provides a potential lever for PE reform. Sport development officers who have represented Fiji possess enormous credibility enabling them to deliver messages that might be dismissed from other sources. Leveraging this credibility to teach about non-communicable diseases prevention, substance use dangers, gender equality could address vulnerability dimensions while maintaining sport engagement.

## Governance: youth exclusion and international inequalities

Youth exclusion from sport governance exemplifies broader patterns of age-based marginalization in Fijian society (25). Despite young people comprising the vast majority of sport participants, they lack voice in decisions affecting programs, resource allocation, and priorities. This reproduces hierarchical structures positioning youth subordinately. As Tisdall (87) observes, the gap between rhetoric and reality in youth participation persists across development sectors, with young people frequently consulted but rarely granted genuine decision-making influence.

Creating youth advisory structures within national sport federations and government agencies would provide formal mechanisms for youth voice. However, meaningful youth participation requires genuine power-sharing where youth influence decisions (23, 88). This challenges traditional authority structures, potentially explaining resistance despite rhetorical commitments to youth development.

International governance inequalities shape Fijian sport profoundly through resource control and rule-making authority concentrated in Global North organizations. Our findings document how AFL Australia and the ICC maintain asymmetric relationships positioning Fiji as a dependent recipient rather than equal partner (40). This mirrors broader patterns in international development where Northern organizations maintain power while ostensibly supporting Southern development (89), (Geeraert, 96). As Houlihan and Zheng (90) demonstrate, international sport governance structures systematically privilege established sporting nations, marginalizing smaller states in resource distribution and rule-making processes.

The non-recognition of informal sport in international funding formulas disadvantages Fijian contexts where significant participation occurs outside formal club structures. Cassidy & Cassidy's (91) documentation of highly organized Lau cricket demonstrates that “informal” sport can be deeply structured and socially significant, yet it receives no international funding recognition. Global North bureaucratic frameworks, requiring registered clubs, formal reporting, centralized governance, privilege their own organizational models while marginalizing different but equally valid forms (92).

## Implications for sport-for-development practice

Our findings yield several implications for sport-for-development practice. First, organizations must move beyond simplistic 'sport as solution' narratives toward critical engagement with how sport reflects and transforms power relations (5, 6). Sport participation doesn't automatically generate positive development outcomes; outcomes depend on program design, governance structures, and contextual factors. Practitioners should assess how programs either challenge or reinforce existing inequalities.

Second, participant-defined priorities must guide programming rather than externally imposed objectives. The SLF's emphasis on individual agency and diverse valued outcomes provides a framework for centering local voices (43, 93). This requires genuine partnerships where communities set agendas rather than simply implementing Northern-designed programs.

Third, gender mainstreaming should be systematic rather than rhetorical (94). This means assessing all policies for gender implications, setting concrete targets for female leadership and resource allocation, monitoring gender-disaggregated outcomes, and challenging masculine organizational cultures.

Fourth, sport-for-development should integrate with broader development sectors rather than operating in isolation (40). Sport intersects with health, education, employment, and social protection systems; effective development requires coordination across these sectors. Current siloed approaches miss opportunities for synergy.

Finally, international sport organizations must acknowledge and address their role in perpetuating Global North dominance. This requires meaningful governance reform giving Southern nations genuine decision-making power, equitable resource distribution including broadcast revenue sharing, and recognition of diverse organizational forms beyond Western club models.

### Limitations

This study has several limitations. First, the cross-sectional design captures participants' perspectives at a single point in time and does not allow assessment of how sport involvement translates into longer-term livelihood trajectories. Second, the findings are based on self-reported experiences and aspirations; reported career intentions and perceived opportunities may not correspond to realised employment or income outcomes. Third, the study is context-specific to Fiji, where labour market structures, migration opportunities, and socio-cultural norms differ from other settings. While this context provides valuable insight into sport–livelihood dynamics in Pacific Island contexts, the findings are not intended to be generalized beyond comparable socio-economic and cultural environments. Furthermore, the focus on AFL and cricket may limit the transferability of findings to other sporting contexts in Fiji, particularly rugby, which occupies a distinctive cultural, social, and economic position within Fijian society.

## Conclusion

This study demonstrates that sport functions as a conditional and gendered livelihood pathway for young people in Fiji. While sport enables the accumulation of social and cultural capital, particularly through networks and the culturally specific concept of *vanua*, these benefits are unevenly distributed and rarely translate into stable economic outcomes. Structural constraints, including limited employment opportunities, unequal access to resources, and fragmented governance systems, shape how and for whom sport generates livelihood opportunities.

Gender emerges as a central axis structuring both participation and outcomes. Female participants face distinct socio-cultural and economic constraints, including domestic responsibilities, limited financial support, and restrictive gender norms, which reduce both access to sport and the benefits derived from it. In contrast, male participants are more likely to convert sport participation into employment opportunities, particularly through pathways linked to national representation. Sport therefore operates not as a neutral development mechanism but as a space where existing gender inequalities are reproduced, albeit with some potential for women empowerment.

These findings highlight the importance of addressing socio-economic constraints on female participation, including through more inclusive governance structures, gender-sensitive resource allocation, and greater integration of youth voices in decision-making. More broadly, the study contributes to understanding how sport intersects with local socio-cultural and institutional contexts to shape unequal livelihood trajectories.

## Data Availability

The raw data supporting the conclusions of this article will be made available by the authors, without undue reservation.
